# Early results of prostate cancer radiation therapy: an analysis with emphasis on research strategies to improve treatment delivery and outcomes

**DOI:** 10.1186/1471-2407-13-23

**Published:** 2013-01-16

**Authors:** Kosj Yamoah, Kwamena Beecham, Sarah E Hegarty, Terry Hyslop, Timothy Showalter, Joel Yarney

**Affiliations:** 1 Department of Radiation Oncology, Kimmel Cancer Center & Jefferson Medical College, Thomas Jefferson University Hospital, Philadelphia, USA; 2 National Center for Radiotherapy and Nuclear Medicine, Korle Bu Teaching Hospital, and College of Health Sciences, University of Ghana, Accra, Ghana; 3 Division of Biostatistics, Department of Pharmacology and Experimental Therapeutics, Thomas Jefferson University, Philadelphia, USA; 4 Department of Radiation Oncology, Thomas Jefferson University Hospital, Philadelphia, PA, USA

**Keywords:** African men, Prostate cancer, External beam RT, Biochemical failure

## Abstract

**Background:**

There is scant data regarding disease presentation and treatment response among black men living in Africa. In this study we evaluate disease presentation and early clinical outcomes among Ghanaian men with prostate cancer treated with external beam radiotherapy (EBRT).

**Methods:**

A total of 379 men with prostate cancer were referred to the National Center for Radiotherapy, Ghana from 2003 to 2009. Data were collected regarding patient-and tumor-related factors such as age, prostate specific antigen (PSA), Gleason score (GS), clinical stage (T), and use of androgen deprivation therapy (ADT). For patients who received EBRT, freedom from biochemical failure (FFbF) was evaluated using the Kaplan-Meier method.

**Results:**

Of 379 patients referred for treatment 69.6% had initial PSA (iPSA) > 20 ng/ml, and median iPSA was 39.0 ng/ml. A total of 128 men, representing 33.8% of the overall cohort, were diagnosed with metastatic disease at time of referral. Among patients with at least 2 years of follow-up after EBRT treatment (n=52; median follow-up time: 38.9 months), 3- and 5-year actuarial FFbF was 73.8% and 65.1% respectively. There was significant association between higher iPSA and GS (8–10 vs. ≤7, p < 0.001), and T stage (T3/4 vs. T1/2, p < 0.001).

**Conclusions:**

This is the largest series reporting on outcomes after prostate cancer treatment in West Africa. That one-third of patients presented with metastatic disease suggests potential need for earlier detection to permit curative-intent therapy. Data from this study will aid in the strategic development of prostate cancer research roadmap in Ghana.

## Background

Prostate cancer is currently the second most often diagnosed cancer and the sixth leading cause of cancer mortality among males worldwide [1]. Incidence rates vary widely among different regions with the highest incidences noted among men from the United States and Europe likely due to utilization of the prostate-specific antigen (PSA) test as a screening tool. Mortality on the other hand is highest among prostate cancer patients of African descent. Based on recent estimates, there is a three-fold higher mortality rate for prostate cancer among patients in African countries as compared to patients in the United States and Europe [2,3]. This trend has been partly attributed to socio-economic factors and inadequate access to healthcare [4,5], as well as differences in genetic susceptibility [6-8].

The available medical literature for prostate cancer is primarily from Europe and the United States. Although the literature does emphasize the outcomes of males of African descent, the subjects included are those who live in developed countries [9-14], which does not represent fully the disease characteristics observed in men who reside in African nations [15,16]. Therefore, there is a need to conduct further studies to better understand and describe prostate cancer in Africa focusing on disease presentation and biochemical failure after currently available treatments, and to develop a roadmap for clinical research aimed at improving treatment delivery and outcomes in Africa.

Reports on cancer patterns among Ghanaian men referred to the Korle Bu Teaching Hospital (KBTH) revealed that prostate cancer comprised 64% of all genitourinary cancers during 1980–1990 [17]. A 10-year retrospective analysis of all cancer deaths at KBTH during 1991–2000, reported by Wiredu and Armah, demonstrated that prostate cancer was the second leading cause of cancer-related mortality among their male patients [18]. Recently, Yarney et al. examined the clinicopathologic features of prostate cancer patients referred to KBTH during 2003–2007, and showed that the majority of 170 patients referred for radiotherapy at KBTH presented with initial PSA >20 ng/ml (73%), Gleason score >7 (56%) and were symptomatic at disease presentation (76%) [19]. This patient profile differs substantially from those encountered in the United States where median initial PSA at diagnosis is estimated at 6.1 ng/ml and 6.3 ng/ml in Caucasians and Black men respectively [20,21].

Clearly, Ghanaian men present with prostate cancer that is more advanced than observed routinely in the United States, and the cancer treatment resources differ dramatically. Our research team plans to develop treatment regimens tailored to the needs of Ghanaian men, which may differ from guidelines currently utilized in the Unites States and Europe in order to better address the disease burden and improve mortality rates in Ghana. In this study we examine early results for definitive radiation therapy for prostate cancer at KBTH, and develop a research roadmap for improving radiation therapy delivery and outcomes in Ghanaian men.

## Methods

### Patient selection

With Institutional Review Board approval from KBTH and Thomas Jefferson University, patient and treatment data were collected retrospectively from the charts of all patients referred to the National Center for Radiotherapy and Nuclear Medicine at KBTH for prostate cancer treatment from January 2003 to December 2009, representing a total of 379 patients. These patients comprise the cohort for evaluation of patient- and tumor-related factors presented in the current report (overall cohort). Among these patients, a total of 251 patients (organ-confined cohort) had non-metastatic disease and were, thus, considered eligible to receive treatment with curative intent consisting of external beam radiotherapy (EBRT) (Cobalt-60 unit), brachytherapy, and/or androgen deprivation therapy (ADT). A subset of this group, the Freedom From biochemical Failure (FFbF) analysis cohort, was identified for evaluation of outcomes after EBRT, with or without ADT (52 patients). Patients who received radical prostatectomy were not included in this analysis, since follow up data were not available. Patients who received radical prostatectomy prior to EBRT or were treated with brachytherapy were excluded from the FFbF analysis cohort, as were patients with radiologic or pathologic evidence of metastatic disease prior to treatment. Inclusion criteria included a biopsy-proven diagnosis of prostate adenocarcinoma, a minimum of 2 years of follow up data, and at least 2 of the following available: clinical T stage, Gleason score, or initial PSA. All patients were initially evaluated by a thorough history, physical examination (including digital rectal examination) followed by routine laboratory studies, bone scan, and serial serum prostate-specific antigen (PSA) during and after treatment. All patients were staged according to the 1992 American Joint Committee on Cancer staging system [22]. Patients were further stratified into low, intermediate and high risk groups according to the recent NCCN guidelines [23].

### Treatment details

#### External beam radiotherapy

Until 2008, prostate EBRT was performed with 2-dimensional treatment planning using a conventional fluoroscopic simulator. A 4-field box technique was applied, with parallel-opposed pairs of antero-posterior and lateral fields, to deliver up to 68- 70 Gy in 34/35 fractions over 7 weeks. The superior border was placed at the lower sacroiliac joint, with the inferior border 1 cm above the bottom of the ischial tuberosities. The anterior border on the lateral film split the pubic symphysis, and posterior border split the S2/S3 vertebrae.

Following the acquisition of a three-dimensional (3D) treatment planning system in 2008, prostate EBRT involves computed tomography (CT) planning. CT scan images were obtained in the supine position with 2.5 mm slice cuts and transmitted to a Prowess Panther Treatment Planning System, version 4.6 (Prowess, Inc., Concord, California). A 3D conformal radiotherapy technique was used. Clinical target volumes (CTV) included the prostate, with or without seminal vesicles. For low risk patients, the CTV included the prostate only, whereas the CTV in intermediate risk patients included the prostate and the inferior 1 cm of the seminal vesicles. The entire seminal vesicles were included in the CTV for high risk disease. Typical margins used to generate a planning target volume (PTV) by expanding the CTV were 1 cm, except posteriorly where the margin was 0.6 cm.

A range of 54-60 Gy was delivered to the initial CTV followed by a second phase, delivering an additional 10 to 14 Gy to the prostate only. Patients received a cumulative central tumour dose of 68 to 74 Gy in 34 to 37 daily fractions over 7 to 7-1/2 weeks. A minimum PTV coverage of 95% coverage was required, with dose inhomogeneity of less than 10% and maximum point dose was not to exceed 107% of prescription dose. Dose-volume histograms (DVHs) were obtained for each patient, with a constraint of V_60_<40% and or V_40_<60% for the rectum. Appropriate shielding with customised blocks was employed to decrease the dose to the rectum and femoral heads. Radiotherapy was delivered using a Cobalt-60 teletherapy (GWGP 80, National Power Institute of China). Portal imaging was obtained from Cobalt 60 machine prior to and midway through treatment course with correction of any set up errors identified.

#### Brachytherapy seed implantation

All patients were seeded using the real-time transrectal ultrasound-guided technique [24]. Iodine-125 sources (Bard Medical Division, Covington, GA, USA) were used in all patients: low risk patients and intermediate risk patients with PSA < 15 ng/ml were prescribed to receive dose 160 Gy (pre-TG-43 formalism), intermediate risk patients with PSA > 15 ng/ml and patients with high risk disease received a partial implant of 110 Gy to the prostate followed by EBRT of 45 Gy to the pelvis.

#### Androgen depravation therapy

ADT consisted of orchiectomy or a gonadotropin-releasing hormone agonist (goserelin acetate) with or without non-steroidal anti-androgen (flutamide or bicalutamide). The use of ADT was dependent on the risk category of the patient. Short term ADT was offered to intermediate risk patients (given only concurrently during the 7–8 weeks of radiotherapy). High risk patients received long term ADT, given for a total of 24 months, beginning 3 months before initiation of radiation [25]. Patients presenting with PSA > 100 ng/ml who completed a negative metastatic work up including bone scintigraphy and CT or magnetic resonance imaging (MRI) of the pelvis were treated as having localized, high-risk disease with EBRT + long term ADT. However, those individuals with PSA > 100 ng/mL and symptoms of new onset bone pain were treated with ADT alone as using EBRT was deemed an exercise in futility due to high likelihood of metastatic disease.

KBTH occasionally receives referrals for consideration of prostate EBRT for patients with rising PSA levels who previously received orchiectomy for localized disease, and who were not assessed at initial diagnosis for radical prostatectomy or radiation therapy. These individuals are evaluated with bone scintigraphy and CT or MRI of the pelvis to determine whether regional or distant spread of prostate cancer is present. Those without clinical or radiological evidence of metastases are then considered for EBRT with curative intent.

### Follow-up and treatment endpoints

All patients in the FFbF analysis cohort, but not the overall cohort, had a follow up of at least two years. Patients were seen in follow up every three months for two years, then every six months for the next three years and once a year subsequently. At each follow up visit, evaluation included DRE and serial PSA values were determined and recorded. When data were available, biochemical failure in the analysis cohort was defined according to the Phoenix definition (PSA nadir + 2 ng/mL). Time to biochemical failure was defined from the end of EBRT. Freedom From biochemical Failure (FFbF) was used as an endpoint in this analysis, since it serves as a surrogate for disease-free survival [26]. Patients with follow up data of less than 24 months after end of EBRT were excluded from FFbF analysis to reduce the influence of long-term ADT on the observed values in the high-risk patients.

### Statistical analysis

Frequency counts and descriptive statistics were used to present information regarding clinical and pathological features of the cohort. Associations between initial PSA level and other factors, including Gleason score, clinical T stage and patient age, were tested using Wilcoxon-Mann–Whitney tests due to the highly skew distribution of PSA levels. For the FFbF Analysis cohort, 3- and 5-year FFbF rates were evaluated using the Kaplan-Meier method. Comparisons of FFbF were performed using the log-rank statistic. The threshold for statistical significance was defined a p-value of <0.05 for all tests. Analyses were performed using the SAS 9.2 statistical software package (SAS Institute Inc., Cary, NC).

## Results

### Patient and disease characteristics

The distribution of the clinical characteristics and treatment modalities for the overall cohort (n=379), the organ-confined cohort (n=251), and the FFbF analysis cohort (n=52) groups are presented in Tables 1 and 2. The median age at diagnosis among Ghanaian men was 65 years. Of all patients referred to KBTH for treatment, 69.6% had initial PSA > 20 ng/mL with a median initial PSA of 39.0 ng/mL. As shown in Table 3, there was a significant association between higher iPSA level and advanced Gleason score (8–10 vs. ≤7, p < 0.001) and clinical T stage (T3/4 vs. T1/2, p < 0.001). There was no significant association between initial PSA and age at presentation (<65 vs. 65+) (p= 0.099). A total of 128 men, representing 33.8% of the overall cohort, were diagnosed with metastatic disease at time of referral and were excluded from further analysis.

**Table 1:** Age and tumor characteristics for prostate cancer patients referred for radiation therapy at Korle Bu teaching hospital during 2003-2009

**Characteristic**	**Overall cohort (n=379)**	**Organ-confined cohort (n=251)**	**FFbF analysis cohort (n=52)**
	**n (%)**	**n (%)**	**n (%)**
**Age, Y**			
<60	78 (20.6)	55 (21.9)	10 (19.2)
60-64	89 (23.5)	66 (26.3)	13 (25.0)
65-69	95 (25.1)	69 (27.5)	17 (32.7)
70-74	76 (20.1)	46 (18.3)	9 (17.3)
>74	41 (10.8)	15 (6.0)	3 (5.8)
**iPSA ng/ml**			
0-10.0	50 (13.2)	46 (18.3)	9 (17.3)
10.1-20.0	50 (13.2)	47 (18.7)	10 (19.2)
20.1-50.0	79 (20.8)	61 (24.3)	16 (30.8)
50.1-100.0	70 (18.5)	45 (17.9)	11 (21.2)
100.1-1000.0	73 (19.3)	40 (15.9)	5 (9.6)
>1000	7 (1.9)	2 (0.8)	1 (1.9)
Unknown	50 (13.2)	10 (4.0)	--
**Gleason Score**			
≤6	130 (34.3)	114 (45.4)	25 (48.1)
7	102 (26.9)	81 (32.3)	20 (38.5)
8 to 10	71 (18.7)	40 (15.9)	5 (9.6)
Unknown	76 (20.1)	16 (6.4)	2 (3.9)
**Clinical T stage**			
T1	17 (4.5)	17 (6.8)	--
T2	114 (30.1)	114 (45.4)	22 (42.3)
≥T3	77 (20.3)	77 (30.7)	29 (55.8)
Tx	43 (11.4)	43 (17.1)	1 (1.9)
M	128 (33.8)	--	--
**Risk Group**			
Low	--	20 (8.0)	4 (7.7)
Intermediate	--	48 (19.1)	9 (17.3)
High	--	166 (66.1)	39 (75.0)
Unknown	--	17 (6.8)	--

**Table 2:** Treatments administered for patients referred for radiation therapy, according to cohort

**Treatment**	**Overall cohort (n=379)**	**Organ-confined cohort (n=251)**	**FFbF analysis cohort (n=52)**
	**n (%)**	**n (%)**	**n (%)**
**EBRT**	147 (38.8)	141 (56.2)	52 (100.0)
EBRT alone	20 (5.3)	18 (7.2)	4 (7.7)
EBRT + ADT	121 (31.9)	117 (46.6)	48 (92.3)
EBRT + Brachytherapy	2 (0.5)	2 (0.8)	--
EBRT+ADT+ Brachytherapy	4 (1.1)	4 (1.6)	--
**ADT alone**	139 (36.7)	60 (23.9)	--
LHRH agonist	120 (31.7)	58 (23.1)	--
Orchiectomy	38 (10.0)	9 (3.6)	--
**Brachytherapy alone**	13 (3.4)	13 (5.2)	--

**Table 3:** Summary of initial PSA (iPSA) levels by various patient characteristics in the overall cohort (n = 379)

**Patient characteristic**	**iPSA ng/ml (quartiles)**			**p-value**
	**25**^**th**^**-percentile**	**Median**	**75**^**th**^**-percentile**	
**Age, Y**				0.099
<65	13.3	32.9	100.0	
≥65	19.9	44.8	100.0	
**Gleason Score**				< 0.001
≤7	13.9	27.9	72.8	
8 to 10	34.6	68.3	268.2	
**Clinical T stage**				< 0.001
T1 - T2	9.4	19.8	39.0	
T3 - T4	22.7	50.4	90.1	

### Treatment characteristics (Organ-confined cohort)

Among 251 patients with organ-confined disease, eligible for definitive radiation therapy (RT), 135 patients (53.8%) received EBRT without brachytherapy at the KBTH (Table 2). Reasons recognized by KBTH clinicians for patients declining EBRT included: the prohibitive cost of treatment, fear of radiation, and a state of denial based on their perception of disease originating solely from spiritual causes rather than biologic processes. Patients treated with EBRT received a median dose of 70 Gy, delivered in 2 Gy daily fractions. Among the organ-confined cohort, 16 patients received orchiectomy, including 7 patients who also received EBRT. At least 214 patients (85%) with organ-confined disease presented with intermediate-to-high risk disease (Table 1). Six patients with low risk prostate cancer were started on ADT by their referring urologist prior to evaluation at KBTH. Seventeen patients in the intermediate- and high-risk groups did not receive ADT during EBRT, due to Gleason score of 5 or less (8 patients) or unaffordable out-of-pocket costs and other socioeconomic factors (9 patients).

### Treatment response (FFbF Analysis cohort)

**Figure 1:**
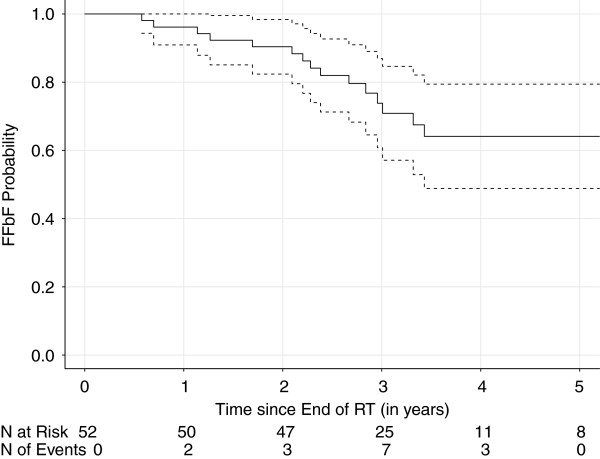
**Kaplan-Meier plot of survival curve of Freedom-From Biochemical Failure (FFbF) for the Analysis Cohort (n=52) of patients who received external beam radiation therapy at Korle Bu Teaching Hospital in Ghana and who were followed for at least 2 years after treatment.** Dotted lines represent 95% confidence interval.

All members of the FFbF analysis cohort (n=52) received EBRT +/− ADT and had at least 2 years of follow up data after treatment (Table 1). Median ADT time was 24 months. Median follow up time in this group was 38.9 months. The 3- and 5-year actuarial FFbF was 73.8% and 65.1% respectively (Figure 1). The 5-year FFbF rates for patients with PSA < 30 ng/mL and PSA > 30 ng/mL were 67.0% and 63.2%, respectively (log-rank, p= 0.586). Acute toxicity during treatment included increased fatigue, urinary frequency, nocturia and bowel frequency, but the rates of these events could not be assessed in the available records. None of the patients on treatment were known to require any surgical intervention or inpatient hospitalization from treatment related causes.

## Discussion

Over the last few decades, there has been a noted increase in the burden of chronic diseases, especially cancer, in Africa [27]. Simultaneously, trends in other factors such as extensive urbanization and lifestyle changes, including smoking, alcohol consumption, and the adaptation of a Western diet, have been linked to an increased risk of cancer [28]. Prostate cancer contributes significantly to these patterns. A recent report on the cancer mortality pattern in Ghana following a 10-year review of autopsies and hospital mortality revealed that prostate cancer was the second leading cause of death from cancer among men in Ghana [18]. Furthermore, the incidence of prostate cancer is on the rise in Ghana, due in part to the fact that the life expectancy of working men has increase over the last decade and better health care facilities have improved detection of disease [19].

The current study describes characteristics of prostate cancer patients referred to KBTH in Ghana and provides insight into early clinical outcomes for the treatment of advanced disease in this population. These data are significant, in terms of defining priorities for cancer care in West Africa, since the majority of the prostate cancer literature originates from the United States and Western Europe and there is an increasing awareness that results obtained from one ethnicity may not necessarily apply to individuals from a different ethnic origin. Over the last two decades, data have emerged from hospital-based cancer registries in a few African countries that provide valuable information. Data from several articles on prostate cancer from 1981 to 2005 indicate an increased prostate cancer risk and mortality among Nigerian men [29-31]. Another study among Senegalese men with prostate cancer revealed worse tumor stage and median PSA when compared with that of African American men [20]. Data from the current study suggest similar findings in our cohort of Ghanaian men. Although this study was done in the largest cancer center in Ghana generalizing this data to all Ghanaian men with prostate cancer must be done with caution. In this section, we will apply these data toward consideration of research priorities aimed at improving prostate cancer diagnosis and treatment in Ghana.

### PSA screening

In our study population of Ghanaian men with prostate cancer, >90% of patients with available data presented with intermediate- or high-risk disease, >95% with clinically T2 or greater disease, and 70% with PSA > 20 ng/ml. In contrast, in the US population, 40-60% of prostate cancer patients present with clinically inapparent disease, mostly diagnosed as T1c upon trans-rectal ultrasound guided (TRUS) biopsy [32,33]. Furthermore, less than 15% of prostate cancer patients in the US population present with PSA > 20 ng/ml [34]. This may be attributed to PSA screening efforts and more frequent TRUS biopsies of prostate in developed countries. Currently, routine yearly PSA screening is a source of controversy in the United States [35], but this approach is not feasible in Ghana where the costs would be prohibitive. Moreover, the effect of PSA screening on prostate cancer mortality in the United States and Europe has been inconclusive. Data from the Prostate Lung Colorectal and Ovarian (PLCO) trial did not show a survival benefit from screening, however the European Randomized Study of Prostate Cancer (ERSPC) trial demonstrated a 31% reduction in the risk of death from prostate cancer in men that had PSA screening [36].

There are major concerns that PSA screening leads to over-diagnosis and overtreatment of indolent prostate cancer in men which if left untreated would have little or no impact on life expectancy [37]. However, in men of African descent who may demonstrate more aggressive disease, the lack of screening could result in an increased number of patients presenting with high risk disease, which would adversely impact prostate cancer mortality rates. This is exemplified in the analysis of our patient cohort showing a strong correlation between PSA levels at diagnosis and advanced clinical T stage as well as Gleason score. Based on trends of prostate cancer mortality in Ghana and the vast majority of patients presenting with high risk disease, it would be advantageous to develop a healthcare policy that will allow for PSA screening along with DRE in a selected cohort of men. Although annual PSA screening would likely exceed financial constraints in Ghana, it may be worthwhile to consider a program that includes less frequent screening. Determining the appropriate initial age for screening and the appropriate time interval for PSA screening in Ghana is beyond the scope of this study, but future studies should address these considerations.

### Challenges to treatment delivery

Out of 251 patients eligible for definitive radiation treatment with curative intent only 141 patients (56.2%) actually received EBRT. A number of factors acting as barriers to treatment delivery include the use of alternative medicines and traditional healing methods coupled with inadequate health education, which often delays correct diagnosis and initiation of treatment. Furthermore, taboos, stigmas, and false beliefs that cancer is a “curse” often lead to delayed diagnosis and non-adherence to treatment. Other barriers specific to radiation treatment delivery among Ghanaian men included fear of radiation, inflated perception of the cost of treatment, difficulty with access to transportation to and from daily treatments, and loss of income due to absence or inability to work.

Ghana has a population of 24 million serviced by only two megavoltage machines in two radiation treatment centers 250 kilometers (180 miles) apart. The lack of accessibility to treatment centers as well as time loss and costs incurred by patient transportation presents a huge barrier for compliance to daily treatments. Furthermore, the national health insurance re-imbursements payment rate to the health care facilities is very low, which in turn renders the out of pocket cost per treatment course per patient enormously expensive for the average working-class Ghanaian man. Currently, shorter course (“hypofractionated”) treatment schedules are being explored for prostate cancer, in an effort to improve patient convenience, reduce costs, and to take advantage of unique radiobiological characteristics of prostate cancer that make large fractions potentially more effective [38]. The adaptation of a hypofractionated schedule for treatment in Ghana would offer a profound advantage in not only decreasing healthcare delivery costs but also improve access to treatment by reducing transportation time and expense for patients during radiation therapy. This represents a potential for implementing tailored prostate cancer treatment schemes for developing countries, an important focus for future studies. To this end, we propose to develop and conduct clinical trials of shorter course radiation therapy schedules tailored to the needs of Ghanaian prostate cancer patients.

### Treatment outcomes

To date, the data presented in this article provides the only source of published information on outcomes for prostate cancer treatment in the West African region. Our results showed that the 3- and 5-year FFbF for Ghanaian men with mostly intermediate to high risk prostate cancer receiving EBRT +/− ADT was 73.8% and 65.1% respectively. In light of differences in patient disease characteristics at diagnosis and older treatment techniques one must consider whether to evaluate these outcomes with respect to the latest published data using dose escalation as reported by Zietman et al. [21] that demonstrated a 80-90% biochemical control as opposed to older experiences from randomized trials such as RTOG 9202 [39] and EORTC 22863 [40] showing biochemical failure rates as high as 50-76% for patients with advanced tumors. A major drawback to this retrospective study is the limited ability to assess important end points such as impact of treatment on cause-specific survival and distant metastases free survival due to a median follow up data of only 3 years. Nevertheless, there is valuable information presented in this article that will aid in the strategic development of a roadmap for prostate cancer research in Ghana, with a focus on improving therapeutic approach as well as fostering a prudent allocation of scarce resources.

### Future research needs

Results presented in this study have demonstrated that the majority of Ghanaian men diagnosed with prostate cancer present with very advanced stage disease. Current treatment recommendations for advanced stage prostate disease are based on clinical trials that include conventionally-fractionated radiation therapy and long-term ADT [39-41]. However, the availability of modern treatment technologies and the more recent interest in hypofractionation for prostate cancer offer an opportunity to develop studies aimed at improving the treatment and outcomes for Ghanaian patients with advanced stage prostate disease. The Ghanaian prostate cancer patient population is in need of clinical trials that seek to develop novel, shorter course treatment regimens for locally-advanced prostate cancer. We have established collaboration between two institutions with the hope of improving prostate cancer treatment in Ghana and plan to develop clinical trials that can be conducted in tandem between our two institutions. Our group encourages approaching the design of clinical trials in a way that includes perspective of the public health burden of prostate cancer in Ghana and the specific barriers to care. We hope to achieve progress by involving stakeholders in a coordinated fashion to develop tailored radiation treatment techniques that are cost-effective and well-suited for the needs of Ghanaian men.

## Conclusion

We have described presentation and early clinical outcomes for a cohort of patients who received prostate cancer treatment at KBTH in Ghana. Based on these results, our group has proposed a plan for future research aimed at identifying an appropriate role for PSA screening in this population, developing radiation therapy treatment schedules that better fulfill the needs of Ghanaian prostate cancer patients, and contributing to understanding genetic factors associated with prostate cancer risk and treatment response.

## Competing interests

The authors have no competing interests to report.

## Authors’ contributions

KY conceived the study, participated in its design including data collection, coordination and helped to draft the manuscript. KB participated in data collection and helped in writing the methods in manuscript. SH performed statistical analysis of data and generated figures in manuscript. TH supervised all statistical analysis and figures in manuscript as well as shaped discussion of findings. TS participated in study design, analysis of data and helped with manuscript writing. JY supervised the entire project and gave access to relevant data for this manuscript, as well as participated in manuscript writing. All authors read and approved the final manuscript.

## Pre-publication history

The pre-publication history for this paper can be accessed here:

http://www.biomedcentral.com/1471-2407/13/23/prepub
